# Genome-Wide Analysis and Expression of *Cyclic Nucleotide–Gated Ion Channel* (*CNGC*) Family Genes under Cold Stress in Mango (*Mangifera indica*)

**DOI:** 10.3390/plants12030592

**Published:** 2023-01-29

**Authors:** Yajie Zhang, Yubo Li, Jing Yang, Xinli Yang, Shengbei Chen, Zhouli Xie, Mingjie Zhang, Yanlei Huang, Jinghong Zhang, Xing Huang

**Affiliations:** 1 Hainan Climate Center, Haikou 570203, China; 2 Environment and Plant Protection Institute, Chinese Academy of Tropical Agricultural Sciences, Haikou 571101, China; 3 College of Tropical Crops, Hainan University, Haikou 570228, China; 4 Guilinyang Campus, Qiongtai Normal University, Haikou 571127, China; 5 Hainan Meteorological Service Center, Haikou 570203, China; 6 School of Life Sciences, Peking University, Beijing 100871, China; 7 College of Plant Science and Technology, Huazhong Agricultural University, Wuhan 430070, China; 8 Key Laboratory of South China Sea Meteorological Disaster Prevention and Mitigation of Hainan Province, Haikou 570203, China; 9 Key Laboratory of Integrated Pest Management on Tropical Crops, Ministry of Agriculture and Rural Affairs, Haikou 571101, China; 10 Hainan Key Laboratory for Monitoring and Control of Tropical Agricultural Pests, Haikou 571101, China

**Keywords:** mango, *CNGC*, phylogeny, cold stress, expression, malondialdehyde

## Abstract

The ‘king of fruits’ mango (*Mangifera indica*) is widely cultivated in tropical areas and has been threatened by frequent extreme cold weather. *Cyclic nucleotide–gated ion channel* (*CNGC*) genes have an important function in the calcium-mediated development and cold response of plants. However, few *CNGC*-related studies are reported in mango, regardless of the mango cold stress response. In this study, we identified 43 *CNGC* genes in mango showing tissue-specific expression patterns. Five *MiCNGCs* display more than 3-fold gene expression induction in the fruit peel and leaf under cold stress. Among these, *MiCNGC9* and *MiCNGC13* are significantly upregulated below 6 °C, suggesting their candidate functions under cold stress. Furthermore, cell membrane integrity was damaged at 2 °C in the mango leaf, as shown by the content of malondialdehyde (MDA), and eight *MiCNGCs* are positively correlated with MDA contents. The high correlation between *MiCNGCs* and MDA implies *MiCNGCs* might regulate cell membrane integrity by regulating MDA content. Together, these findings provide a valuable guideline for the functional characterization of *CNGC* genes and will benefit future studies related to cold stress and calcium transport in mango.

## 1. Introduction

The *cyclic nucleotide–gated ion channel* (*CNGC*) family belongs to nonselective cation channels, which enable the uptake of ions, including K^+^, Ca^2+^ and Na^+^ [1]. The channel gate-control function of *CNGC* genes confers their essential roles in regulating plant growth and development [2]. Furthermore, members of the *CNGC* family are reported to mediate cellular ion homeostasis to regulate abiotic and biotic stress response [3]. Usually, plant *CNGC* genes contain six transmembrane domains, in which the cyclic nucleotide-binding domain (CNBD) is between the fifth and sixth transmembrane domains [4]. The CNBD domain is highly conserved and could be used to identify *CNGC* genes among plant species [5]. To date, the *CNGC* family has been widely reported in many plant species, such as in *Arabidopsis thaliana* (20), *Atalantia buxfolia* (31), *Brassica oleracea* (26), *Brassica rapa* (29), *Citrus grandis* (30), *Citrus recticulata* (27), *Citrus sinensis* (32), *Gossypium hirsutum* (40), *Gossypium barbadense* (41), *Gossypium herbaceum* (20), *Gossypium arboreum* (20), *Gossypium raimondii* (20), *Nicotiana tabacum* (35), *Oryza sativa* (16), *Poncirus trifoliata* (30), *Pyrus bretchneideri* (21), *Solanum tuberosum* (20), *Triticum aestivum* (47), *Zea mays* (12) and *Ziziphus jujuba* (15) [2,5,6,7]. Most importantly, their functions have been well characterized in model plant *Arabidopsis*. For example, 15 *AtCNGCs* (*AtCNGC1-10*/*12*/*14*/*16*/*18*/*20*) are encoded Ca^2+^-permeable channels [8], and numerous studies confirm their functions in plant development, such as *AtCNGC2* in leaf senescence, *AtCNGC3* in germination, *AtCNGC5*/*6*/*9* in root hair development, *AtCNGC7*/*8* in male fertility, *AtCNGC14* in root gravitropism and *AtCNGC16*/*18* in pollen development [9,10,11,12,13,14,15]. Several *AtCNGCs* are involved in stress response, including *AtCNGC1*/*10*/*19*/*20* in salt stress, *AtCNGC2*/*4*/*11*/*12* in abiotic stress, *AtCNGC2*/*5*/*6*/*9*/*12* in stomatal defense and *AtCNGC6* in heat stress [16,17,18,19,20,21,22]. Together, these findings provide valuable references for functional characterization and application of *CNGC* genes in non-model plant species.

Mango, known as the ‘king of fruits’, is one of the most popular fruits [23]. Its annual fruit yield ranks fifth around the world [24]. The main cultivated *Mangifera* species in the tropical areas around the world is *Mangifera indica* [25]. As a typical tropical plant, mango is sensitive to cold temperature [26], especially frequent extreme cold weather, which has significantly threatened to mango production in recent years [27]. However, few studies have revealed the molecular basis of cold stress response in mango trees, even if the cold storage of detached fruits has been well studied [28]. Considering that overexpression of *CNGC* genes promotes rice cold tolerance, we conducted genome-wide analysis of the *CNGC* family in mango and evaluated its expression in mango tissues, as well as that under cold stress [29]. The results showed that expression of several *MiCNGCs* was upregulated under cold temperature and highly correlated to leaf damage index malondialdehyde (MDA) contents, implying their beneficial roles in regulating mango cold tolerance. Therefore, our study will offer guidance for functional characterization of *CNGC* genes and benefit future studies in mango.

## 2. Results

### 2.1. Characterisation of CNGC Family in Mango

**Figure 1:**
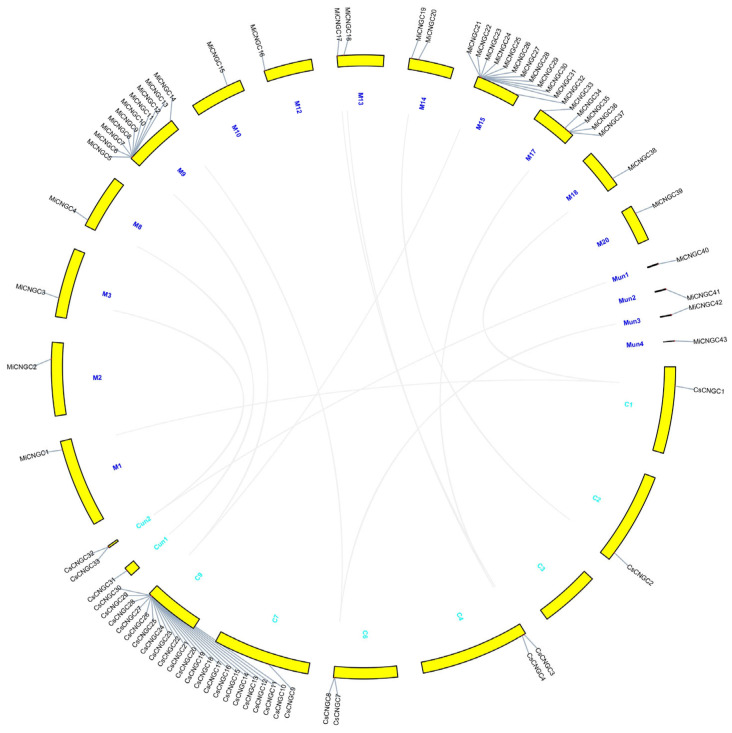
Chromosome distribution of *CNGC* genes in mango (blue) and sweet orange (light blue). The chromosome numbers are marked with numbers beside the chromosomes. Homologous gene pairs between the two species are linked with lines. The ‘un’ represents unanchored scaffolds.

The *Arabidopsis* and rice *CNGC* genes were selected to search homologous genes in the genomes of *Amborella trichopoda*, sweet orange (*Citrus sinensis*) and mango [24,30,31]. Because sweet orange belongs to the Sapindales order together with mango, it was selected as a nearby reference [24]. Amborella was chosen as the outgroup reference to the Sapindales order since it is the basal angiosperm [30]. As a result, 7, 33 and 43 *CNGC* genes were identified in above three species (Table 1 and Table S1). In mango species, these genes ranged from 399 to 2343 bp with predicted protein lengths of 132–780 aa. Their molecular weights and theoretical pI ranged from 14907.27 to 89883.31 Da and 4.88 to 9.67, respectively. Most of them (36 of 43) were predicted to be located at the plasma membrane, contain 3–7 transmembrane helices (Figure S1). Five and two were predicted to be nuclear and extracellular, respectively. There was no more than one transmembrane helix in these seven genes. A total of 39 mango *CNGC* genes were distributed at 13 chromosomes. Two major gene clusters, including 10 and 12 *CNGC* genes at chromosomes 9 and 15, respectively, were labeled (Figure 1).

### 2.2. Phylogenetic Relationships of Mango CNGC Genes

**Figure 2:**
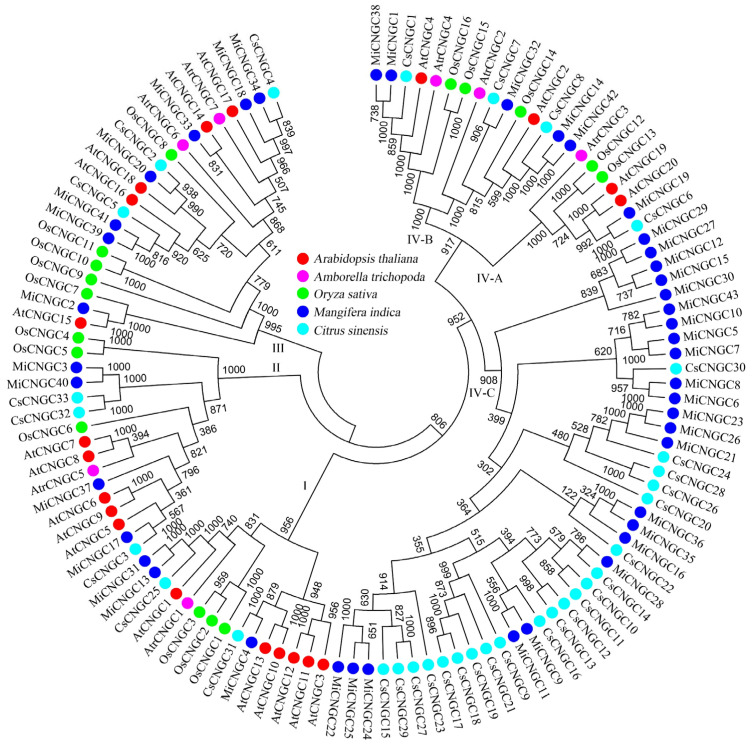
Maximum-likelihood phylogenetic tree of CNGC proteins in *Arabidopsis thaliana* (red), *Amborella trichopoda* (pink), *Oryza sativa* (green), *Mangifera indica* (blue) and *Citrus sinensis* (light blue).

The CNGC proteins of *Arabidopsis*, rice, Amborella, sweet orange and mango were selected to construct the maximum-likelihood phylogenetic tree (Figure 2). Finally, four groups (I–IV) were generated, where almost the same quantities of CNGC proteins from the four species existed in each group. Interestingly, only sweet orange and mango CNGC proteins were clustered into group IV-C. Mango CNGC proteins were further aligned to identify their conserved domains. The results showed that most of them contained the CNBD domain ([L]-X(0,1,2)-[G]-X(1,3)-G-X(1,2)-[L]-[L]-X(0,1)-[W]–X(0,2)-[L]–X (0,7,8,9,10,18)-[P]-X-S-X(10)-[E]-A-[F]-X(0,1)-L) except *MiCNGC43* (Figure S2). However, nine mango CNGC proteins lost the N-termini due to evolution issues, namely *MiCNGC10*, *11*, *15*, *21*, *25*, *27*, *28*, *35* and *36*. We further calculated the values of synonymous substitutions (Ks), nonsynonymous site (Ka) and their ratio (Ka/Ks). The results indicated that all the Ka/Ks values were below 1 in homologous gene pairs (Table S2).

### 2.3. In Silico Expression of CNGC Genes in Mango Tissues

**Figure 3:**
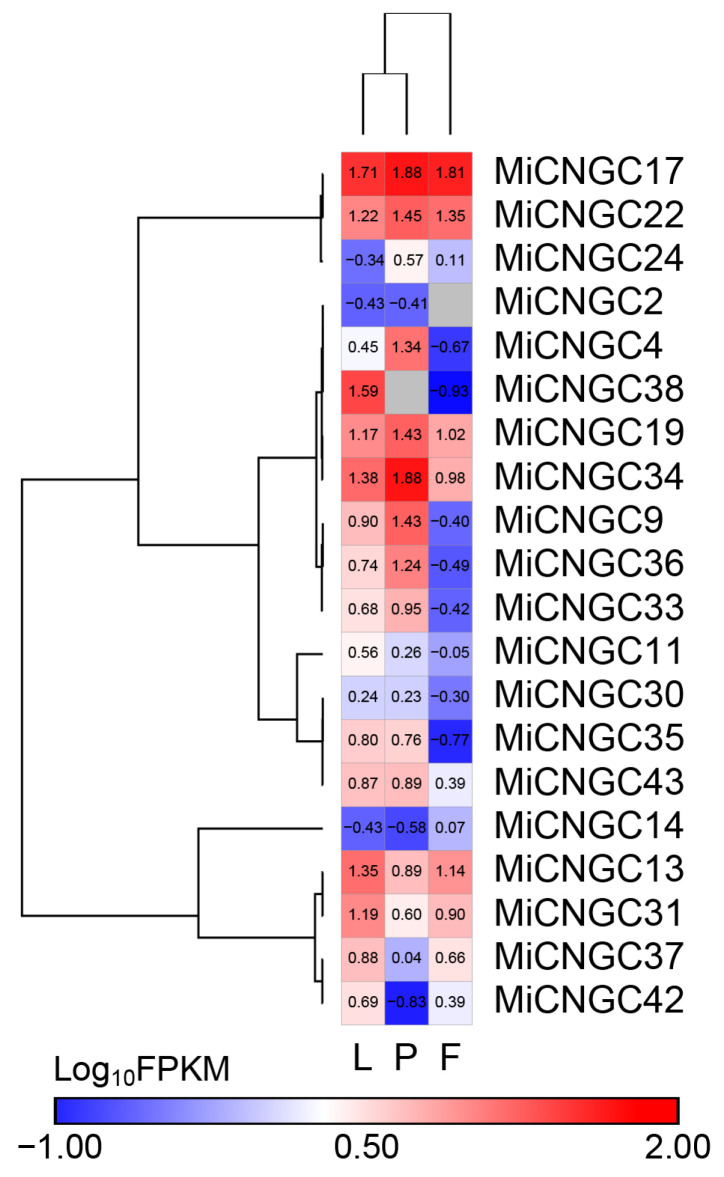
In silico expression of mango *CNGC* genes in different tissues, including leaf (L), fruit peel (P) and fruit flesh (F).

We further examined the expression levels of mango *CNGC* genes in leaf, fruit peel and fruit flesh based on published transcriptome data. The expression data were normalized into fragments per kilobase of exon model per million mapped fragments (FPKM) (Table S3). *MiCNGC17*, *19* and *22* were relatively expressed at high levels in all three tissues (FPKM > 10) (Figure 3). Several tissue-specific expressed mango *CNGC* genes were revealed too, such as *MiCNGC13*, *31* and *38* in leaf and *MiCNGC4*, *9*, *36* and *34* in fruit peel. At the same time, 10 mango *CNGC* genes displayed extremely low expression (FPKM < 10) in all three tissues, namely *MiCNGC2*, *11*, *14*, *24*, *30*, *33*, *35*, *37*, *42* and *43*, whereas 23 genes showed no expression detected by transcriptome sequencing (Table S3).

### 2.4. In Silico Expression of CNGC Genes under Cold Stress in Mango Fruit Peel

**Figure 4:**
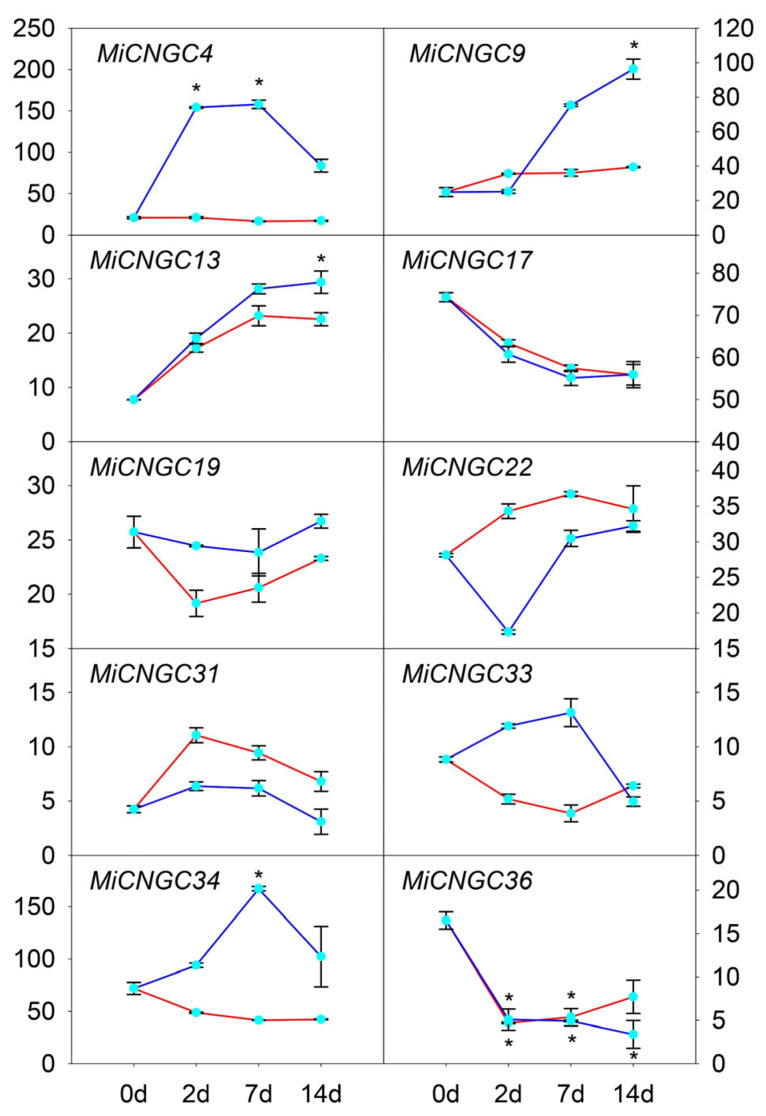
In silico expression of *CNGC* genes under cold stress of (blue) and 12 °C (red) in mango fruit peel. The *x*-axis represents the samples collected at 0, 2, 7 and 14 days after treatment. The *y*-axis represents FPKM values. The error bar represents the standard error. * represents that the expression level was up or downregulated over 3-fold.

In order to evaluate their molecular functions under cold stress in fruit peel, moderate (12 °C) and extreme (5 °C) temperatures were chosen to compare *CNGC* expression patterns. The two treatments allow us to better understand the expression tendency of *CNGC* genes under cold stress. The results indicated that most *CNGC* genes (33 of 43) showed no or relatively low expression levels (FPKM < 10) under cold treatments of both 5 and 12 °C, implying that they might play minimal roles for cold tolerance (Table S3). However, 10 *CNGC* genes showed higher expression triggered by cold stress (Figure 4). Among these, three of them showed moderate expression patterns under 12 °C, but more than three-fold upregulated expression level under 5 °C (*MiCNGC4*, *9* and *34*). Five genes showed similar expression patterns whether at 5 or 12 °C (*MiCNGC13*, *17*, *19*, *31* and *36*), while *MiCNGC13* and *36* were up and downregulated over 3-fold under 5 °C after prolonged 14-day cold treatment, respectively. Moreover, *MiCNGC22* and *MiCNGC33* showed absolutely opposite expression patterns under the two degrees.

### 2.5. Expression Profiles of CNGC Genes under Cold Stress in Mango Leaf

**Figure 5:**
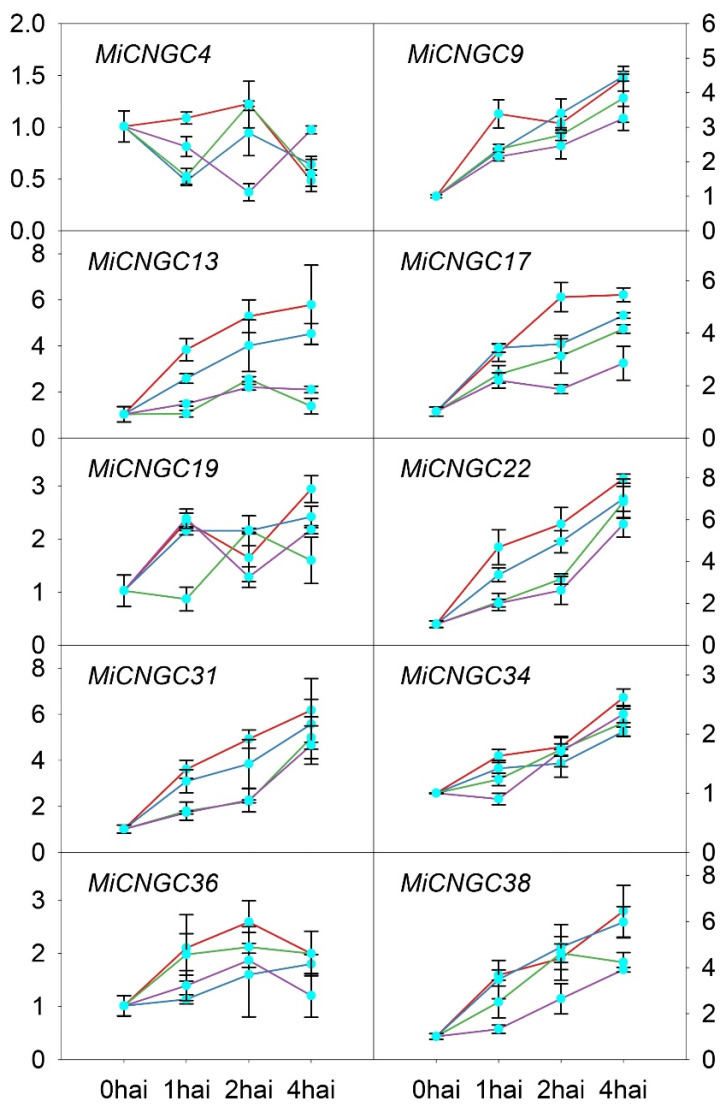
The expression of *CNGC* genes under cold stress of 2 (red), 4 (blue), 6 (green) and 8 °C (purple) in mango leaf according to qRT-PCR. The *x*-axis represents the samples collected at 0, 1, 2 and 4 h after incubation (hai) at different temperatures. The *y*-axis represents the values of 2^−ΔΔCt^. The error bar represents the standard error.

To better understand how the *CNGC* genes affect cold stress response in mango plant, 10 mango *CNGC* genes were selected for qRT-PCR validation in mango leaf. These genes include five with expression changes over threefold under cold stress in fruit peel and five with FPKM values over 10 in leaf. The results indicated that most genes were upregulated after cold treatment in leaf except *MiCNGC4* (Figure 5). Among these, six were up-regulated more than threefold, namely *MiCNGC9*, *13*, *17*, *22*, *31* and *38*. Additionally, the expression of *MiCNGC13* was not significantly affected when temperature was higher than 4 °C, indicating that *MiCNGC13* might have leaf- and fruit-peel-specific temperature sensitivity.

### 2.6. Positively Correlated Mango CNGC Genes with MDA Contents

**Figure 6:**
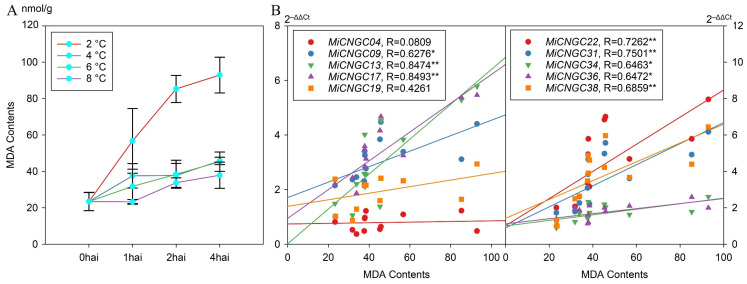
The MDA contents under cold stress of 2 (red), 4 (blue), 6 (green) and 8 °C (purple) in mango leaf (**A**) and their correlations with the relative expression levels of *CNGC* genes (**B**). * and ** represent that the correlation coefficients are significant at 0.05 (R > 0.532) and 0.01 (R > 0.661) levels, respectively.

The content of malondialdehyde (MDA) is usually considered as a lipid peroxidation index that indicates the damage of stress. We therefore further measured MDA in mango leaf to evaluate the physiological effects of cold stress. As expected, MDA contents were significantly increased under cold treatment, specially under 2 °C (Figure 6A). This result suggested that temperatures under 2 °C might cause irreversible damage to the mango plant. To further determine whether *CNGC* genes regulate MDA level or not, we performed correlation analysis to explore *CNGC* genes that were highly correlated with MDA contents. The results showed that three and five *CNGC* genes were positively correlated with MDA contents with significant correlation coefficients at 0.05 (R > 0.532) and 0.01 (R > 0.661) cut-offs, respectively.

## 3. Discussion

### 3.1. Species-Specific Expansion of CNGC Family in Mango

In the present study, we have successfully identified 7, 33 and 43 *CNGC* genes in Amborella, sweet orange and mango (Table 1 and Table S1). As a basal angiosperm, Amborella has a relatively small number of *CNGC* genes compared with other four species (Figure 2). Beyond this, these genes are almost evenly distributed into three groups (I, II and III) and two subgroups (IV-A and IV-B), indicating the conserved evolution patterns and pressure among these groups [4]. Interestingly, there is a subgroup (IV-C) containing *CNGC* genes from either mango (23) or sweet orange (21). Most of them are distributed at chromosomes 9 and 15 in mango (18) and chromosome 9 in sweet orange (21) (Figure 1), verifying their Sapindales classification. However, the species-specific expansion of *CNGC* genes among mango and sweet orange also emphasizes the species divergence during evolution [32]. In addition, mango *CNGC* genes are classified into two major gene clusters, which might be divided from the same cluster during the hypothetical auto-diploidization [24]. Seven mango *CNGC* genes contain short coding regions (<1000 bp), such as *MiCNGC10*, *MiCNGC28* and so on (Table 1), which might be caused by frequent chromosomal recombination (Table S3) [33]. They might lose the function of Ca^2+^-permeable channels without complete transmembrane structure (Figure S1), which also affected their subcellular localizations predicted by CELLO.

### 3.2. Candidate CNGC Genes in Cold Stress Response in Mango

The in silico expression of *CNGC* genes in mango tissues illustrates that mango *CNGC* genes have tissue-specific expression patterns (Figure 3). The high and constitutive expression of *MiCNGC17* indicate that it might affect the whole mango development period. We further evaluated the expression of *CNGC* genes under cold stress to identify candidate regulators that contribute to cold tolerance. As shown, five *MiCNGCs* in fruit peel and five in leaf displayed more than three-fold upregulation of gene expression; specifically, *MiCNGC9* and *MiCNGC13* are induced in both tested tissues suggesting their potential functions under cold stress (Figure 4 and Figure 5). Since MDA is the main indicator of cell membrane integrity [34], we observed that MDA content is significantly increased at 2 °C compared with 4, 6 and 8 °C (Figure 6A), which means that lower temperature leads higher damage. Then we revealed that eight *MiCNGCs* are positively correlated with MDA by correlation analysis (Figure 6B), emphasizing that *MiCNGCs* might regulate MDA content to adjust cell membrane integrity. Therefore, these eight genes could be considered as early cold-responsive markers in the mango leaf, among which *MiCNGC13* ranks first as the early cold-responsive maker gene in the mango leaf and fruit peel.

## 4. Materials and Methods

### 4.1. Bioinformatic Analysis of CNGC Genes

CNGC protein sequences of *Arabidopsis* and rice were selected as queries to search homologous proteins using the Blastp method [4,35]. The genomes of Amborella, sweet orange and mango were set as targets for sequence retrieval [24,30,31]. The details of *CNGC* genes in Ambrella and sweet orange are listed in Table S1. The accession numbers and chromosome positions of mango *CNGC* genes are in Table 1 and were further used to illustrate their distribution in chromosomes using TBtools software [36]. The sequence features of length, molecular weight and pI were predicted using the ProtParam tool with subcellular localization predicted using CELLO software [37,38]. The TMHMM2.0 software was selected to predict the transmembrane helices in *CNGC* proteins [39]. A maximum-likelihood phylogenetic tree was constructed for phylogenetic analysis with bootstrap values of 1000 using MEGA 7.0 software [40]. The mango CNGC proteins were further aligned by DNAMAN7 software to examine the conserved domains [41]. The Ks, Ka and Ka/Ks values were calculated using TBtools software [36].

### 4.2. In Silico Gene Expression Analysis

The raw data of transcriptome sequencing were downloaded from the Sequence Read Archive (SRA) database [42]. The raw data of leaf (SRR3288569), fruit peel (SRR2960401) and fruit flesh (SRR11060165) were selected to evaluate the expression patterns of *CNGC* genes in different mango tissues. The raw data of cold-treated fruit peel (SRP066658) were selected to evaluate the expression patterns of *CNGC* genes in mango fruit peels under cold stress, which were generated at 0, 2, 7 and 14 days after storage at 5 and 12 °C [43]. All raw data were trimmed to generate clean reads, which were further mapped to mango *CNGC* genes to calculate FPKM values for each gene with the RSEM software [44,45]. The heat map was generated using Morpheus software [46]. The hierarchical clustering method was selected to cluster the FPKM values at the levels of tissues and genes [47].

### 4.3. Plant Materials and Treatment

The mango variety Hongyu was selected for cold treatment. Experiments were carried out in a mango garden (109.11° E, 19.22° N) at Jishi Village, Changjiang, China. The branches of 15-year-old trees were put into RR-CTC806C incubators (Rainroot Scientific, Beijing, China) for cold treatments at four temperatures, namely 2, 4, 6 and 8 °C. After that, leaves were collected at 0, 1, 2 and 4 h. Leaves from two branches were put together as one sample and each sample was repeated three times as a biological replicate. All the samples were stored at −80 °C before further analysis.

### 4.4. Quantitative PCR and MDA Assay

Total RNA was isolated from each sample using the Tiangen RNA prep Pure Plant Kit (Tiangen Biomart, Beijing, China) and then transcribed into cDNA using the GoScript Reverse Transcription System (Promega, Madison, WI, USA) [48]. Quantitative PCR was conducted with the QuantStudio 6 Flex Real-Time PCR System (Thermo Fisher Scientific, Waltham, MA, USA). The reaction program contained three stages of initiation (94 °C for 30 s), 40 cycles (94 °C for 5 s and 60 °C for 30 s) and dissociation. The TransStart Tip Green qPCR SuperMix (Transgen Biotech, Beijing, China) was selected as the reaction solution, containing 10 μL supermix, 0.4 μL Passive Reference Dye, 1 μL cDNA, 0.5 μL of two primers and 7.6 μL nuclease-free water. Each sample was repeated three times as technical replicates. The Primer 3 software was used for primer design, and *MiActin* was selected as a reference gene (Table 2) [49]. The relative expression levels were calculated with the ΔΔCt method as previously described [50,51]. The MDA contents were examined using the Malondialdehyde (MDA) Content Assay Kit (Solarbio, Beijing, China) according to the manufacturer’s instruction [52]. About 0.1 g of each sample was broken into powder in liquid nitrogen and isolated with 1 mL Extraction reagent, which was fully homogenized in ice and centrifuged for supernatant (8000× *g* for 10 min at 4 °C). A total of 100 μL supernatant was mixed with 300 μL MDA working reagent and 100 μL Reagent III. Distilled water was selected as a blank reference. The mixtures of samples (T) and distilled water (B) were incubated for 60 min under 100 °C, which were cooled and centrifuged (10,000× *g* for 10 min) for supernatant at room temperature. The supernatants of mixtures (200 μL) were placed in a 96-well flat-bottom plate for the detection of absorbance (A) at 450, 532 and 600 nm with the Biotek Synergy H1 system (Agilent Technologies, Lexington, MA, USA). The MDA content was calculated with the following formula, MDA (nmol/g) = (12.9 × (∆A532 − ∆A600) − 1.12 × ∆A450) × Vrv ÷ (W × Vs ÷ Vsv) = 5 × (12.9 × (∆A532 − ∆A600) − 1.12 × ∆A450) ÷ W. Vrv, Vs, Vsv and W represent total reaction volume (0.5 mL), sample volume (0.1 mL), the volume of Extraction reagent (1 mL) and sample weight, respectively. ∆A450 = A450(T) − A450(B), ∆A532 = A532(T) − A532(B), ∆A600 = A600(T) − A600(B).

## 5. Conclusions

Extreme cold weather is a significant threat to the tropical fruit tree mango. Thus, we carried out a genome-wide analysis and assessed the expression of mango *CNGC* genes under cold stress, due to their importance in calcium-mediated development and cold response. The result revealed 43 *CNGC* genes with species-specific expansion in the mango genome. In silico expression analysis indicated their tissue-specific expression patterns and five differentially expressed *CNGC* genes under cold stress in mango fruit peel. The results of the qRT-PCR validation and MDA assay revealed five differentially expressed *CNGC* genes under cold stress in the mango leaf, which are also positively correlated with MDA contents. These results indicate a candidate early cold-responsive marker gene, *MiCNGC13*, in the mango leaf and fruit peel, which will be helpful to future studies related to cold stress in mango.

## Figures and Tables

**Table 1:** Characterization of mango *CNGC* genes, including their accession numbers, chromosome positions, the lengths of coding sequences and predicted proteins, pI and subcellular locations.

Gene ID	Accession Number	Chromosome Position	Coding Sequence (bp)	Predicted Protein (aa)	Molecular Weight (Da)	Theoretical pI	Subcellular Localization
*MiCNGC1*	LOC123217317	Chr1: 26617757–26622513 (+)	2088	695	80,238.87	8.59	Plasma Membrane (4.159)
*MiCNGC2*	LOC123202006	Chr2: 18877379–18881267 (−)	2115	704	80,732.55	9.33	Plasma Membrane (4.168)
*MiCNGC3*	LOC123211819	Chr3: 6181866–6187338 (+)	2298	765	88,428.88	9.17	Plasma Membrane (3.621)
*MiCNGC4*	LOC123223438	Chr8: 1942929–1947032 (+)	2151	716	82,987.45	9.38	Plasma Membrane (3.546)
*MiCNGC5*	LOC123224898	Chr9: 570085–574702 (−)	1734	577	67,095.52	9.67	Plasma Membrane (3.860)
*MiCNGC6*	LOC123225620	Chr9: 590058–593780 (−)	1203	400	46,407.24	9.12	Plasma Membrane (2.575)
*MiCNGC7*	LOC123225300	Chr9: 599306–603542 (+)	1998	665	77,653.8	9.42	Plasma Membrane (3.398)
*MiCNGC8*	LOC123225621	Chr9: 608291–611565 (+)	1377	458	53,035.69	9.52	Plasma Membrane (2.835)
*MiCNGC9*	LOC123225476	Chr9: 613450–633947 (−)	1821	606	69,498.22	5.61	Plasma Membrane (3.921)
*MiCNGC10*	LOC123225622	Chr9: 613655–614273 (+)	516	171	19,510.66	4.88	Nuclear (2.027)
*MiCNGC11*	LOC123225623	Chr9: 640657–644088 (−)	1260	419	48,200.04	5.71	Plasma Membrane (2.541)
*MiCNGC12*	LOC123226204	Chr9: 654424–660087 (−)	1731	576	66,396.96	8.51	Plasma Membrane (3.243)
*MiCNGC13*	LOC123225899	Chr9: 662666–667252 (−)	2130	709	81,778.71	9.18	Plasma Membrane (4.474)
*MiCNGC14*	LOC123224819	Chr9: 17603913–17607726 (−)	2151	716	82,061.84	9.54	Plasma Membrane (4.714)
*MiCNGC15*	LOC123226709	Chr10: 13110947–13113872 (+)	1023	340	38,771.01	9.24	Plasma Membrane (2.866)
*MiCNGC16*	LOC123193626	Chr12: 337616–340675 (−)	1635	544	61,685.37	8.3	Plasma Membrane (3.945)
*MiCNGC17*	LOC123195239	Chr13: 132576–141356 (+)	2208	735	83,845.52	9.06	Plasma Membrane (4.347)
*MiCNGC18*	LOC123195166	Chr13: 2244118–2249930 (+)	2193	730	84,150.34	8.66	Plasma Membrane (4.075)
*MiCNGC19*	LOC123196387	Chr14: 571224–582588 (−)	2343	780	89,883.31	9.05	Plasma Membrane (4.810)
*MiCNGC20*	LOC123196103	Chr14: 2913165–2916467 (+)	2148	715	82,156.57	8.92	Plasma Membrane (4.612)
*MiCNGC21*	LOC123198417	Chr15: 882187–884231 (+)	729	242	28,545.32	9.19	Nuclear (1.618)
*MiCNGC22*	LOC123198080	Chr15: 884570–888789 (+)	1716	571	66,348.13	6.17	Plasma Membrane (3.842)
*MiCNGC23*	LOC123198017	Chr15: 894806–897058 (+)	1266	421	48,693.95	5.97	Plasma Membrane (4.227)
*MiCNGC24*	LOC123197882	Chr15: 899688–903479 (+)	1809	602	70,104.71	8.73	Plasma Membrane (4.512)
*MiCNGC25*	LOC123198019	Chr15: 937711–939887 (−)	744	247	28,653.79	8.06	Nuclear (1.582)
*MiCNGC26*	LOC123198301	Chr15: 944971–947978 (−)	1563	520	59,757.67	8.86	Plasma Membrane (3.850)
*MiCNGC27*	LOC123197889	Chr15: 957259–959253 (−)	708	235	27,110.7	9.57	Extracellular (1.327)
*MiCNGC28*	LOC123197890	Chr15: 968337–969069 (−)	399	132	14,907.27	8.37	Extracellular (1.999)
*MiCNGC29*	LOC123197893	Chr15: 998581–1002107 (−)	1524	507	58,308.01	8.81	Plasma Membrane (4.361)
*MiCNGC30*	LOC123197894	Chr15: 1002388–1013277 (−)	1926	641	74,391.25	8.83	Plasma Membrane (4.326)
*MiCNGC31*	LOC123198006	Chr15: 1014910–1019679 (−)	2130	709	81,816.51	9.13	Plasma Membrane (4.548)
*MiCNGC32*	LOC123197621	Chr15: 13323057–13327587 (−)	2028	675	77,023.97	9.41	Plasma Membrane (4.667)
*MiCNGC33*	LOC123199977	Chr17: 659050–663360 (−)	2163	720	83,381.02	8.99	Plasma Membrane (4.360)
*MiCNGC34*	LOC123200530	Chr17: 10040631–10047558 (+)	2178	725	83,488.05	9.09	Plasma Membrane (4.053)
*MiCNGC35*	LOC123201033	Chr17: 11813705–11815638 (−)	696	231	26,664.04	9.05	Nuclear (1.519)
*MiCNGC36*	LOC123200218	Chr17: 11846751–11849774 (−)	690	229	26,194.33	8.47	Nuclear (1.544)
*MiCNGC37*	LOC123200982	Chr17: 12162919–12170581 (+)	2136	711	81,639.43	9.03	Plasma Membrane (4.349)
*MiCNGC38*	LOC123201393	Chr18: 10879862–10886175 (+)	2061	686	79,312.97	9.09	Plasma Membrane (4.272)
*MiCNGC39*	LOC123204873	Chr20: 2710625–2713311 (+)	2052	683	79,142.16	9	Plasma Membrane (4.212)
*MiCNGC40*	LOC123206450	NW_025401129.1: 250730–254202 (−)	2229	742	85,358.83	9.37	Plasma Membrane (3.988)
*MiCNGC41*	LOC123206532	NW_025401132.1: 147597–150404 (+)	2052	683	79,185.18	9.05	Plasma Membrane (4.213)
*MiCNGC42*	LOC123206927	NW_025401145.1: 36673–40482 (+)	2151	716	82,061.84	9.54	Plasma Membrane (4.714)
*MiCNGC43*	LOC123208231	NW_025401260.1: 224–2797 (−)	1176	391	44,867.26	9.23	Plasma Membrane (3.815)

**Table 2:** Primers used for qRT-PCR analysis.

Genes	Forward Primer	Reverse Primer	Product Size (bp)	Accession Number
*MiCNGC4*	TTTACTGCTTCTGGTGGGGT	AGGGAGCAAACGATGAGACA	236	XM_044646600.1
*MiCNGC9*	TCAGCTTCCTCGTTGACCTT	CTTCCCGCTTCCAACATCAG	226	XM_044649431.1
*MiCNGC13*	TCGGGCTTCAGGATTCTTGT	CCCAGTCCACCTCTTCATGT	196	XM_044650230.1
*MiCNGC17*	CTTCAAACGAGCACCTTCCC	TCCTCGTGTTTCCAACCACT	246	XM_044608894.1
*MiCNGC19*	ACTTGGGAATGTCAGGAGCA	CCAACAACATGACCAGCCAA	193	XM_044610397.1
*MiCNGC22*	TCACATGGGCTCTAGATGGC	AAACAGAGTCATCCGGCAGA	175	XM_044612662.1
*MiCNGC31*	GACTTGGGCAGCTTGTTTCA	TGAATCCTTGCCTTCCGAGT	216	XM_044612575.1
*MiCNGC34*	CGCCGTTTTGACCAGTACAA	AGCATCTCGGTTACAGGGTC	248	XM_044615727.1
*MiCNGC36*	GCAAGACAGAGCAGTGGATG	TCCTCTAATGCCGATTCGCT	232	XM_044615370.1
*MiCNGC38*	ATTCTCCCTCTCCCTCAGGT	TGTGCCGAAAATGTAGCCAG	183	XM_044616876.1
*MiActin*	CCACTGCTGAACGGGAAAT	GTGATGGCTGGAAGAGGAC	192	HQ585999.1

## Data Availability

All data are contained within the article or Supplementary Materials.

## Supplementary Materials

The following supporting information can be downloaded at: https://www.mdpi.com/article/10.3390/plants12030592/s1, Figure S1: Transmembrane topology analysis for mango *CNGC* proteins; Figure S2: Alignment of *CNGC* proteins in mango; Table S1: Details of *CNGC* genes in amborella and sweet orange; Table S2: The values of synonymous substitutions (Ks), nonsynonymous site (Ka) and their ratio (Ka/Ks) in homologous gene pairs.; Table S3: FPKM values of mango *CNGC* genes in different tissues and under cold stress.
