# Supplementary figures and images for: Genome-Wide Analysis and Expression of Cyclic Nucleotide–Gated Ion Channel (CNGC) Family Genes under Cold Stress in Mango (Mangifera indica)
Source: Plants (Basel). 2023 Jan 29;12(3):592. doi: 10.3390/plants12030592 (PMC9920709; doi:10.3390/plants12030592)

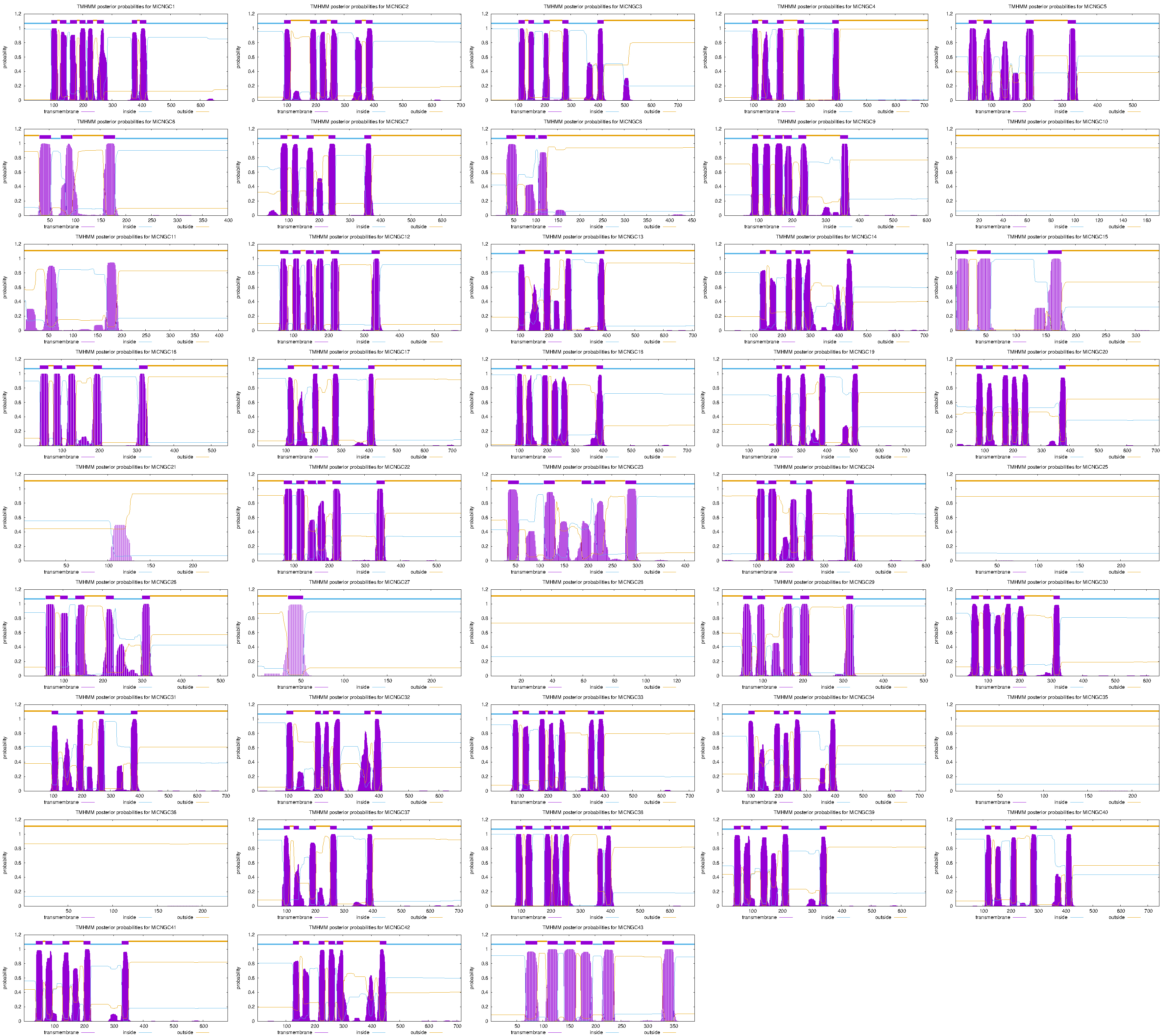

Supplement: Supplementary file 1 [file plants-12-00592-s001.zip › Figure S1.jpg]
